# Co/Cd-MOF-Derived Porous Carbon Materials for Moxifloxacin Adsorption from Aqueous Solutions

**DOI:** 10.3390/molecules29163873

**Published:** 2024-08-15

**Authors:** Fuhua Wei, Xue Gong, Qinhui Ren, Hongliang Chen, Yutao Zhang, Zhao Liang

**Affiliations:** 1 College of Chemistry and Chemical Engineering, Anshun University, Anshun 561000, China; 18812374451@163.com (X.G.); rqh285554560@163.com (Q.R.); chenhledu@126.com (H.C.); zyt0516@126.com (Y.Z.); 2 Institute of Micro/Nano Materials and Devices, Ningbo University of Technology, Ningbo 315211, China

**Keywords:** antibiotics, adsorption, wastewater treatment

## Abstract

In this study, Co/Cd-MOFs were synthesized via a solvothermal method. The resulting material was subjected to calcination at 900 °C for 2 h and characterized using FT-IR, XRD, and SEM techniques to assess its efficacy in moxifloxacin removal. The experimental findings revealed that the maximum adsorption capacity of Co/Cd-MOFs for moxifloxacin was observed at 350.4 mg/g within a 5 h timeframe. Furthermore, the analysis based on the pseudo-second-order kinetic model demonstrated that the adsorption process adhered to this specific model. Additionally, the adsorption isotherm analysis indicated that Freundlich multilayer adsorption provided the best description of the interaction between moxifloxacin and the Co/Cd-MOF material. These experimental and theoretical results collectively suggest that employing Co/Cd-MOFs as adsorbents holds promise for wastewater treatment applications.

## 1. Introduction

The rapid growth of human society has led to increased environmental pollution, particularly due to widespread industrialization and urbanization [1,2,3]. The latest statistics reveal that the global population witnessed a surge from 6.4 billion to 7.7 billion between 2003 and 2019; based on this trajectory, it is projected to surpass 10 billion by 2050 [4,5]. It has been reported that approximately 30% of the world’s population lacks access to potable water [6]. Antibiotics are extensively used in medical treatment, agriculture, and aquaculture because of their strong ability to kill or inhibit bacteria. According to a recent study conducted by Mulchandani et al., it is projected that the global annual usage of antibiotics in livestock will reach approximately 107,500 tons by 2030, representing an increase from just under 100,000 tons in 2020. Notably, Asia, particularly China, exhibits the highest utilization of antibiotics. Furthermore, there is an anticipated growth of antibiotic consumption by an additional 8% between 2020 and 2030. However, this excessive use has resulted in significant water contamination issues in China [7]. The detection rates of antibiotics in soil, surface water, and coastal waters were found to be 100%, 98.0%, and 96.4%, respectively. One study revealed that human and livestock feces constituted the primary sources of pharmaceutical antibiotics and veterinary antibiotic emissions, accounting for 57.6% and 42.6% of the total emissions, respectively. Pig manure accounted for a significant proportion (98.7%) of antibiotic residues in livestock manure, as predicted by the antibiotic report. In the Pearl River Basin, amoxicillin exhibited the highest concentration at 3384 ng/l followed by fluprofen (2867 ng/L), while other antibiotics such as norfloxacin and penicillin showed concentrations exceeding 1000 ng/L. Various methods are currently employed for removing antibiotics from water, including biological photocatalysis [8], treatment [9], filtration [10], coagulation/flocculation [11], chemical precipitation [12], membrane treatment [13], ion exchange [14], and adsorption techniques [15]. Among these approaches, adsorption is favored due to its simplicity in design, cost-effectiveness, high efficiency, convenient operation, insensitivity to toxic substances, and recyclability. Carbon materials with large specific surface area, high porosity, and high reactivity levels have been widely utilized as effective adsorbents for eliminating antibiotics from aquatic environments [16]. Adsorbents like activated carbon nanotubes and graphene materials can efficiently remove numerous pollutants without worsening the existing pollution levels [17,18]. However, common carbon materials often face limitations such as small pore size and low adsorption capacity [19]. Therefore, MOFs (Metal–organic Frameworks) have attracted considerable attention for pollutant removal.

Despite the numerous advantages of MOFs, they also have certain limitations, such as their limited solubility in water and poor stability at high temperatures. As a result, their industrial application is still relatively low. MOFs are mainly used in various applications including sensing [20], separation [21], storage [22,23], imaging [24], and catalysis [25]. In recent years, the utilization of MOF adsorption for the removal of antimicrobials has emerged as a prominent area of research, encompassing notable examples such as ZIF-8 [26,27], MOF-5/ILG [28], and Zr-MOFs [29]. On the other hand, porous carbon materials have become widely popular due to their large surface area, cost-effective preparation methods, and easy regeneration [30]. Moreover, the hydrophobic nature of porous carbon greatly reduces water molecule adsorption. By using MOFs as precursor systems for synthesizing porous carbon materials, we can overcome some disadvantages associated with the poor water and thermal stability of MOFs while retaining their inherent advantages. Currently available methods for synthesizing multi-porous carbon materials based on MOFs include direct carbonization, additional carbon source carbonization, and chemical activation techniques [31]. For example, porous carbon materials can be prepared by directly converting MIL-100 (Al) or MOF-5 into porous carbons [32,33]. Additionally, Zn/Co bimetallic ZIF materials can be directly converted into a series of porous carbons at different temperatures [34]. The utilization of MOFs as precursor systems simplifies the process and eliminates the need for template agents while providing new guidelines for preparing and applying such materials [35]. Derived from MOF precursors, the resulting porous carbons show great potential in the remediation of organic pollutants.

Various preparation methods exist for carbon materials, with the main techniques for porous carbon materials including template synthesis, activation processes, and polymer-based approaches. To overcome these technical limitations, various strategies using structures such as mesoporous, hollow, yolk–shell, multidimensional hollow, or porous structures have emerged to facilitate the investigation of nanostructured carbon-based materials [36]. In this study, the Co/Cd-MOF material was synthesized by employing 1,3,5-benzoic acid as an organic ligand and cadmium acetate dihydrate along with cobalt chloride hexahydrate as sources of metal ions. The Co/Cd-MOF material was subjected to calcination at 900 °C for 2 h to obtain carbonaceous material, which was subsequently utilized for the removal of antibiotics.

## 2. Results and Discussion

The Co/Cd-MOF materials were synthesized using solvothermal methods and characterized through various techniques such as FT-IR, XRD, and SEM. Furthermore, the carbonized Co/Cd-MOF materials were utilized for the removal of moxifloxacin. Subsequently, simulation studies were conducted which involved analyzing a pseudo-first-order kinetic model, a pseudo-second-order kinetic model, and performing isothermal adsorption experiments.

### 2.1. Structural Characterization of Co/Cd-MOF Materials

**Figure 1:**
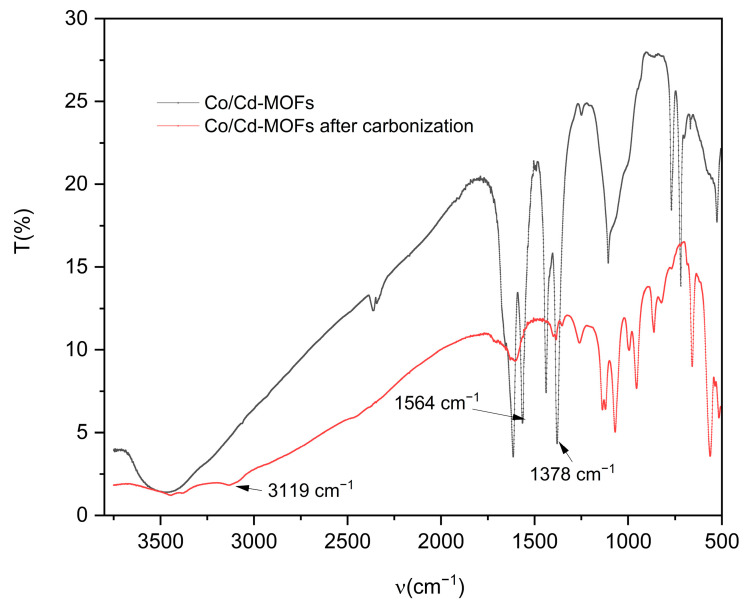
FT-IR of Co/Cd-MOFs before and after carbonization.

**Figure 2:**
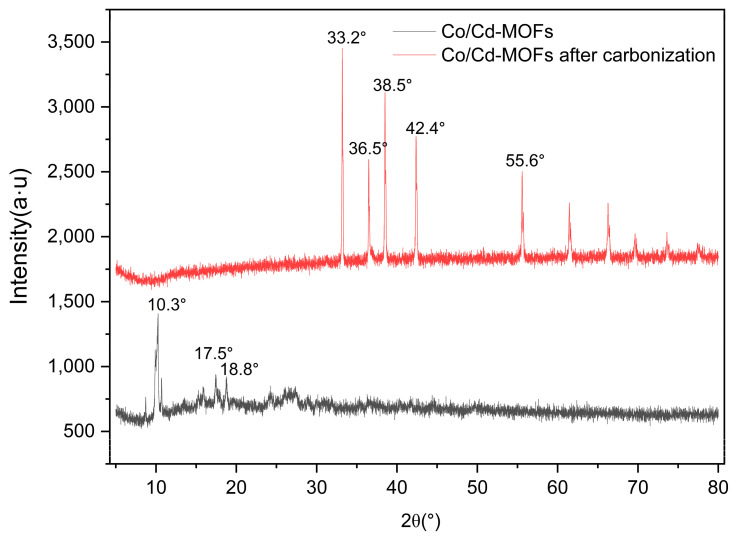
XRD of Co/Cd-MOFs before and after carbonization.

**Figure 3:**
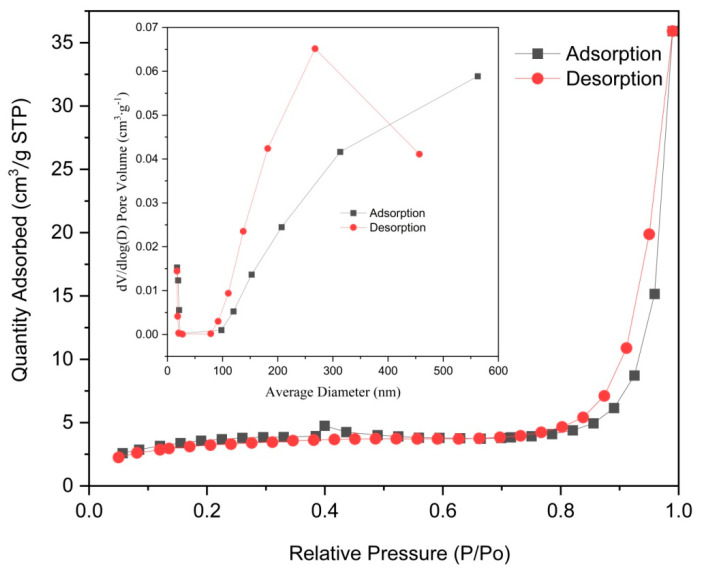
N_2_ adsorption–desorption isotherms of Co/Cd–MOFs.

**Figure 4:**
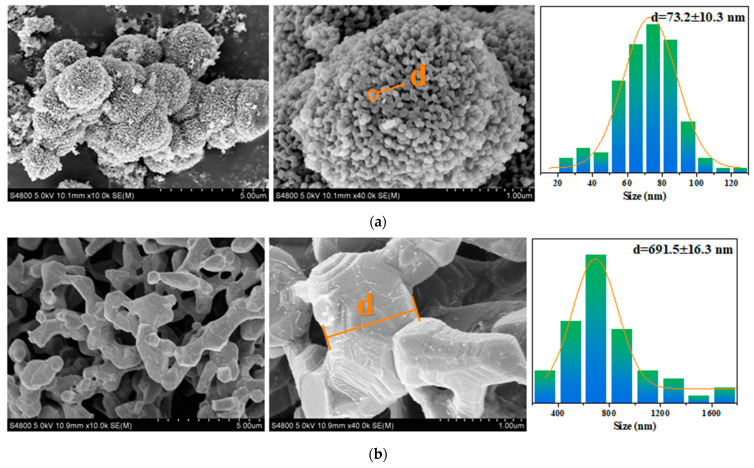
SEM of Co/Cd-MOFs before and after carbonization ((**a**), precarbonization; (**b**), carbon compound).

The FT-IR analysis (Figure 1) reveals prominent absorption peaks at 1565 and 1379 cm^−1^ for Co/Cd-MOFs, which can be attributed to the formation of two equivalent C-O bonds resulting from the delocalization of carboxyl groups [37,38]. The wide weak peak at 3119 cm^−1^ primarily arises from the stretching vibration of the C-H bond. The disappearance of characteristic peaks related to C-H and carboxyl groups in the benzene ring after the carbonization of Co/Cd-MOFs is evident from Figure 1. Notably, significant changes occur in the 2θ absorption peak of Co/Cd-MOFs before and after carbonization, as depicted in Figure 2. Furthermore, the analysis of nitrogen adsorption and desorption isotherms presented in Figure 3 reveals a measured BET specific surface area value measuring approximately 12.33 m^2^/g. The interaction between the adsorbed material and gas appears relatively weak as molecules tend to accumulate around regions with higher attractiveness on the surface. Furthermore, the SEM observations (Figure 4) confirm that excellent dispersion is achieved upon the carbonization of Co/Cd-MOFs.

During the carbonization process, the coordination bond between the organic ligand and metal ions of MOF materials is disrupted, leading to the restructuring of the material’s framework. This structural transformation induces alterations in the crystal structure of MOFs, consequently impacting their XRD profile. Following carbonization, novel diffraction peaks may emerge in the XRD pattern due to the formation of amorphous carbon. Simultaneously, the carbonization of MOF materials also induces modifications in their infrared spectra. The chemical bonds within organic ligands break and recombine during this process, resulting in the disappearance of characteristic peaks observed in infrared spectra. These changes reflect alterations in the chemical structure of MOF materials during carbonization. During the carbonization process, surface morphology alterations such as changes in pore structure and particle size can occur on MOF materials which will be evident from SEM images after carbonization.

### 2.2. Elimination of Moxifloxacin Using Carbonized Materials Derived from Co/Cd-MOFs

**Figure 5:**
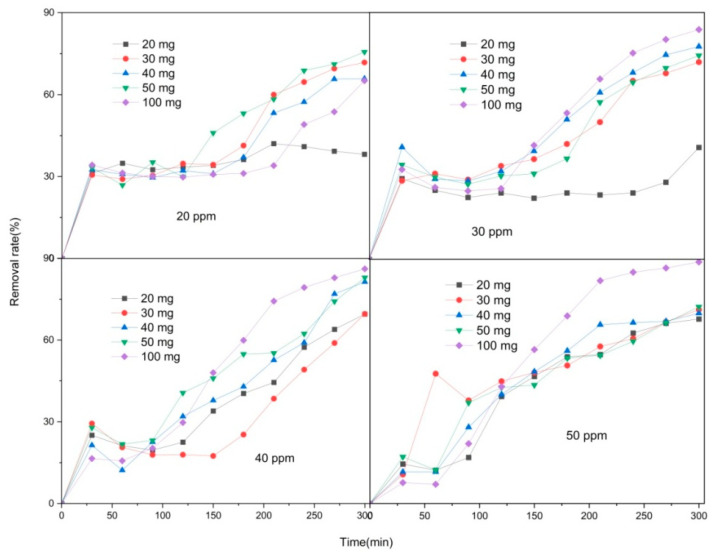
Removal rate of Co/Cd-MOF carbon material to moxifloxacin.

**Figure 6:**
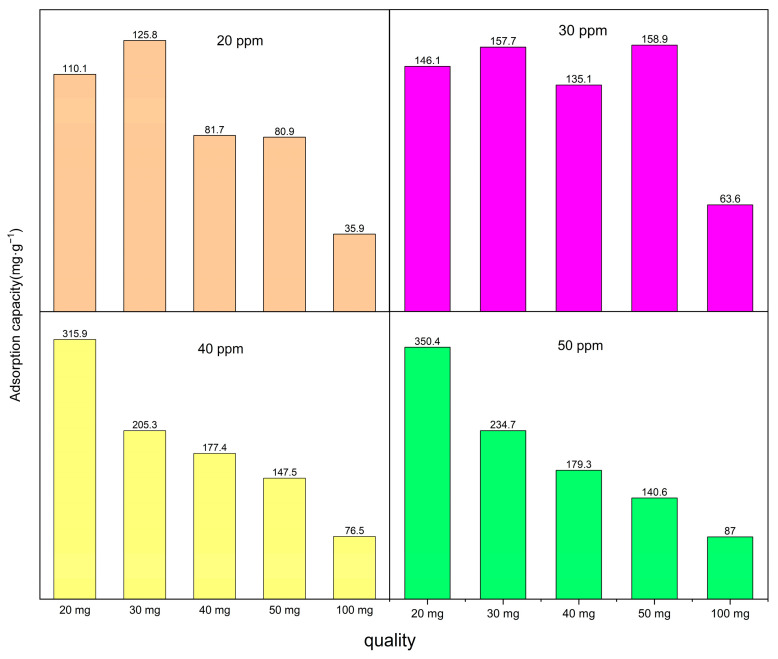
Adsorption capacity of Co/Cd-MOF carbon material to moxifloxacin.

**Figure 7:**
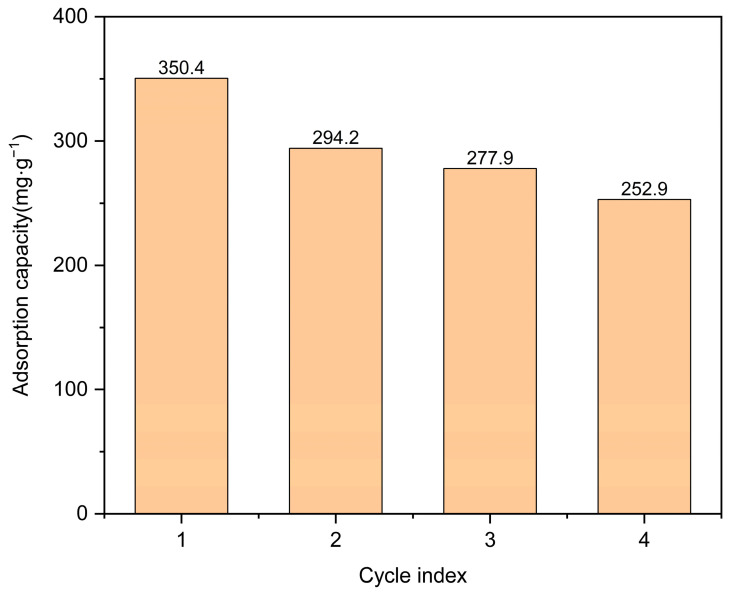
Cyclic diagram illustrating adsorption of moxifloxacin by Co/Cd-MOF carbon material.

This study investigated the characteristics of carbonized Co/Cd-MOF materials and their effectiveness in removing moxifloxacin, a targeted pollutant. The adsorption time, amount of Co/Cd-MOF carbon materials, and concentration of moxifloxacin solution were systematically manipulated for thorough exploration. As shown in Figure 5, when using 100 mg of Co/Cd-MOF carbon material and a moxifloxacin solution with a concentration of 50 ppm, an impressive removal rate of 88.9% was achieved within a span of 5 h. Additionally, the experimental results indicated that increasing the quantity of the Co/Cd-MOF carbon material under constant moxifloxacin concentration conditions resulted in higher removal rates due to the enhanced exposure and involvement of active sites during the adsorption process. Conversely, when keeping the quantity of the Co/Cd-MOF carbon material constant, lower concentrations for moxifloxacin solutions corresponded with a decreasing trend in the removal rate as more molecules competed for each active site during the adsorption process. Moreover, by reducing the amount of the Co/Cd-MOF carbon material to 20 mg while maintaining the same concentration for the moxifloxacin solution, an exceptional adsorption capacity reaching up to 350.4 mg/g within the same timeframe was observed on the Co/Cd-MOF carbon material surface (as shown Figure 6). The adsorption capacity can be enhanced by improving the quality of the adsorbent, as this will provide a greater number of active sites for adsorption, thereby increasing the likelihood of successful adsorption. Additionally, an increase in the mass of the adsorbent allows for a higher quantity of molecules to be adsorbed, resulting in an increased overall amount of adsorption. Furthermore, the concentration of the material being absorbed also influences the extent of adsorption. When there is a higher concentration of absorbate present, more interactions occur between absorbate molecules and the surface of the adsorbent, leading to an augmented level of adsorption. The adsorption capacity of the adsorbent is limited, and upon reaching a certain concentration level, the adsorption sites become saturated, resulting in no further increase in the adsorption amount. As shown in Figure 7, following four cycles, the sample exhibits an impressive adsorption capacity of 252.9 mg/g, demonstrating the recyclability of the Co/Cd-MOF carbon material for moxifloxacin removal. This distinction primarily lies in the disparity between chemisorption and physical adsorption, as well as their respective characteristics. In contrast, chemisorption arises from the chemical bonding forces between the adsorbate and adsorbent, typically resulting in monolayer adsorption. This necessitates specific activation energy and exhibits notable selectivity.

The adsorption process of moxifloxacin onto Co/Cd-MOF carbon materials is accurately described using the pseudo-first-order and pseudo-second-order kinetic models. By simulating these two models based on experimental data, our aim is to evaluate the level of agreement between theory and practice, as well as gain insights into the adsorption mechanism of moxifloxacin on Co/Cd-MOF carbon materials. The dynamic formula employed for this purpose is presented below [39].

(1) $$\ln\;C_{t}/C_{0}=k_{1}t$$

(2) $$\frac{t}{q_{t}}=\frac{t}{q_{e}}+\frac{1}{k_{2}q_{e}^{2}}$$

In these equations, C_t_ represents the concentration of moxifloxacin at a given time point t; C_0_ denotes its initial concentration; k_1_ and k_2_ are rate constants in units of minutes that govern this kinetic reaction; and t signifies the duration of this reaction in minutes. Additionally, q_t_ refers to the adsorbent’s concentration (measured in mg/g) at a specific time point t, whereas q_e_ indicates its equilibrium state.

**Figure 8:**
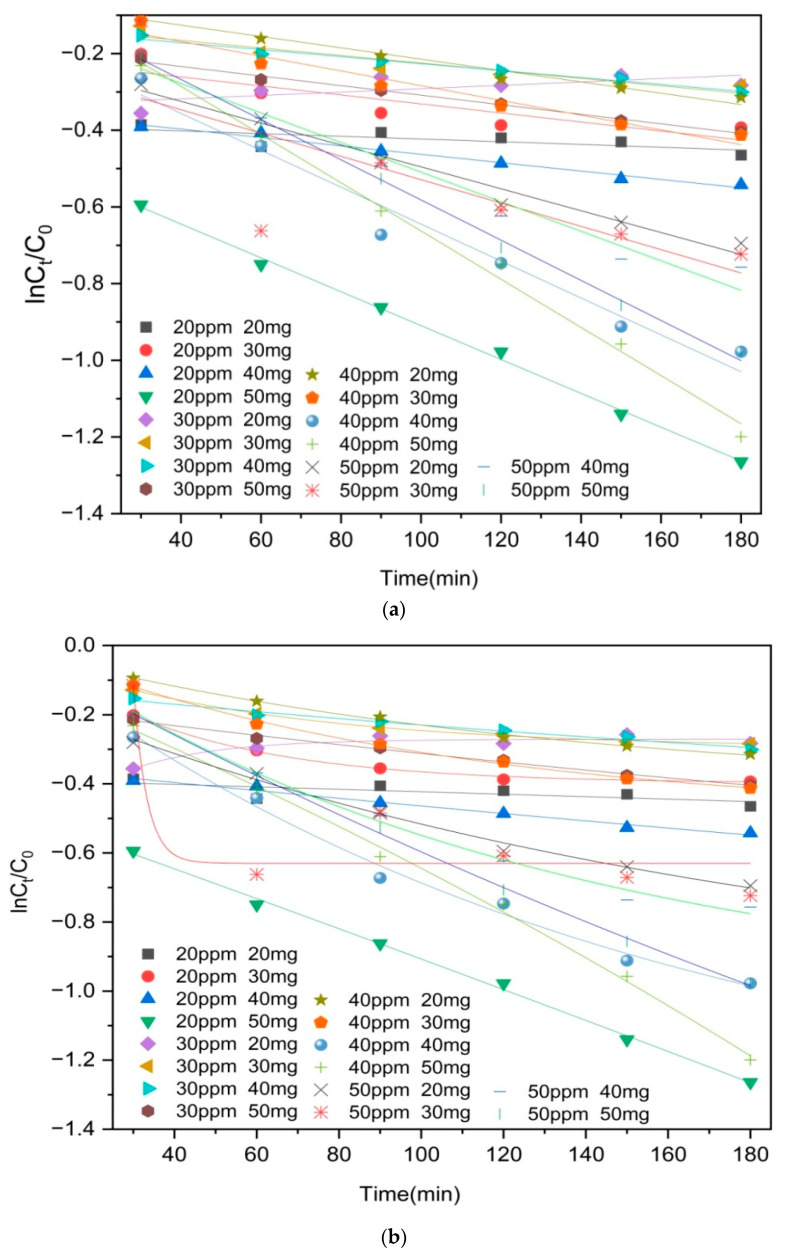
Pseudo-first-order kinetic model of moxifloxacin adsorption by Co/Cd-MOF carbon materials ((**a**). linear; (**b**). nonlinear).

**Figure 9:**
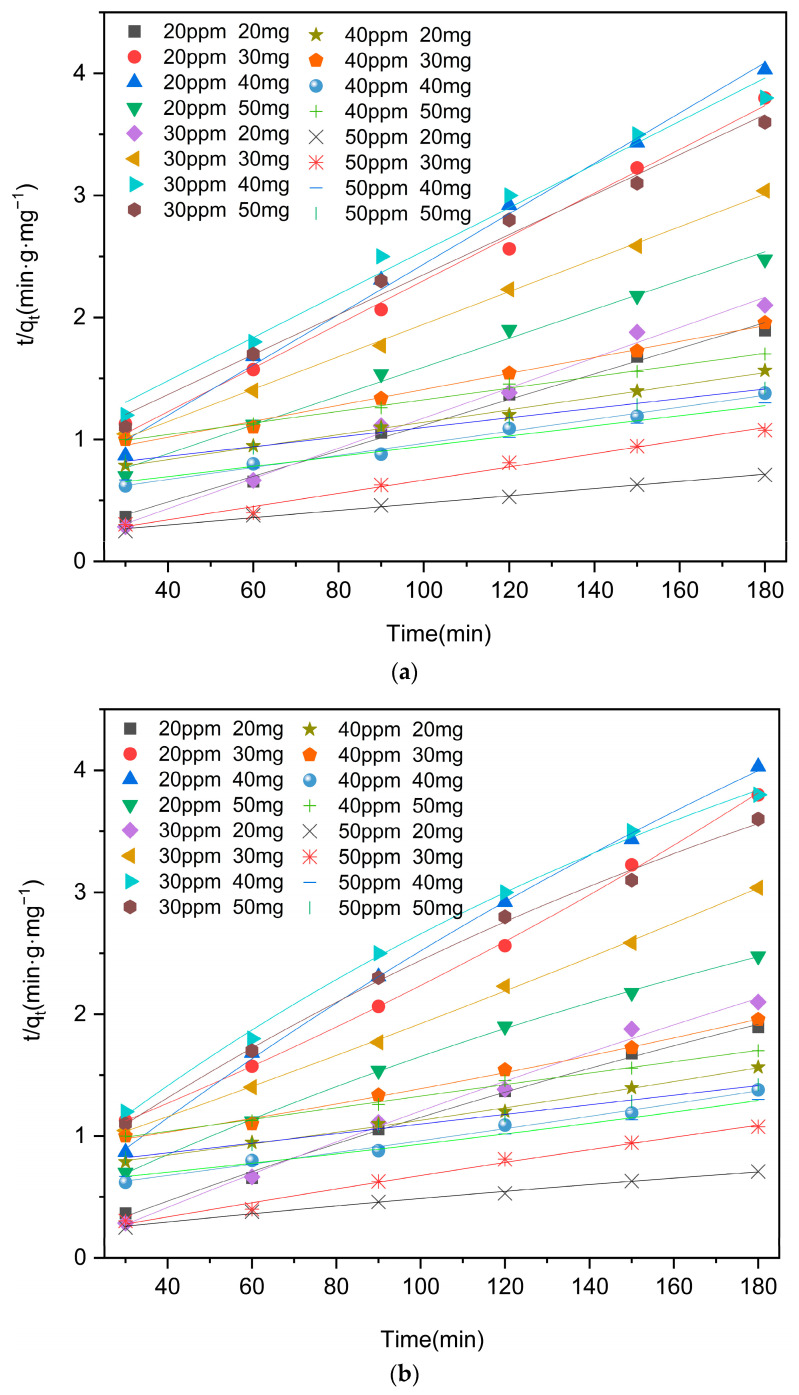
Pseudo-second-order kinetic model of moxifloxacin adsorption by Co/Cd-MOF carbon materials ((**a**). linear; (**b**). nonlinear).

The results of the linear and nonlinear analyses based on the pseudo-first-order kinetic and pseudo-second-order kinetic models are presented in Figure 8 and Figure 9 and Table 1 and Table 2. It is noteworthy that the R^2^ values obtained from the nonlinear second-order kinetic models outperform those derived from the pseudo-first-order kinetic models, indicating that the degradation of moxifloxacin using Co/Cd-MOF carbonized materials can be effectively described by the pseudo-second-order kinetic model. The adsorption of moxifloxacin by Co/Cd-MOF carbonized materials is primarily governed by the surface abundance of active sites on the adsorbent and the strength of interaction between the adsorbent and moxifloxacin.

**Figure 10:**
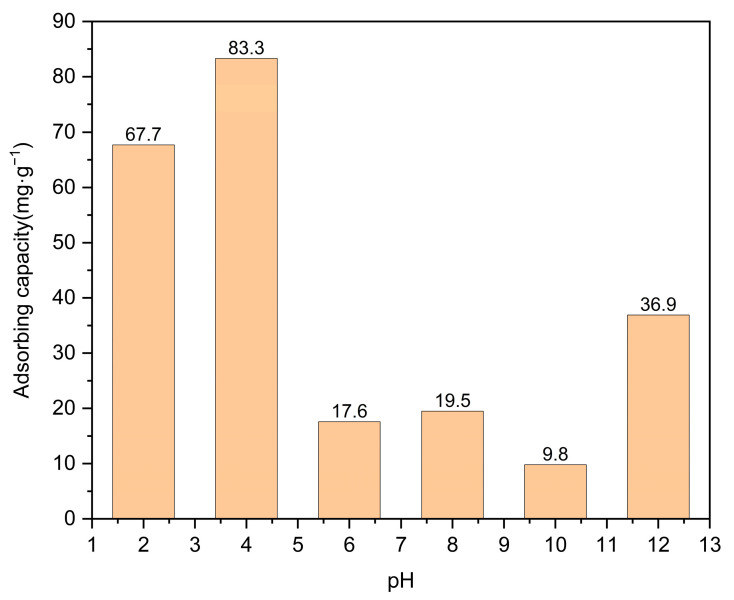
Effect of pH on adsorption of moxifloxacin by Co/Cd-MOF carbon materials.

To investigate the influence of solution pH on moxifloxacin adsorption, a 50 ppm moxifloxacin solution at pH levels ranging from 2 to 12 was introduced to Co/Cd-MOF-incorporated carbonized materials with a mass of 20 mg. After three hours of stirring, the results presented in Figure 10 revealed that optimal moxifloxacin adsorption occurred at pH 4, primarily attributed to charge effects resulting in enhanced substance adsorption per unit mass or volume.

**Figure 11:**
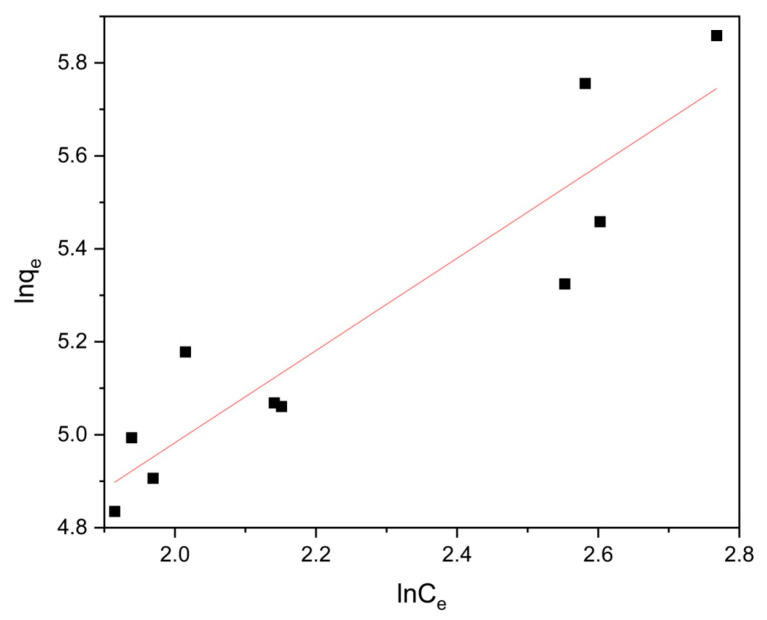
Freundlich isotherm of moxifloxacin onto Co/Cd-MOF carbon material.

**Figure 12:**
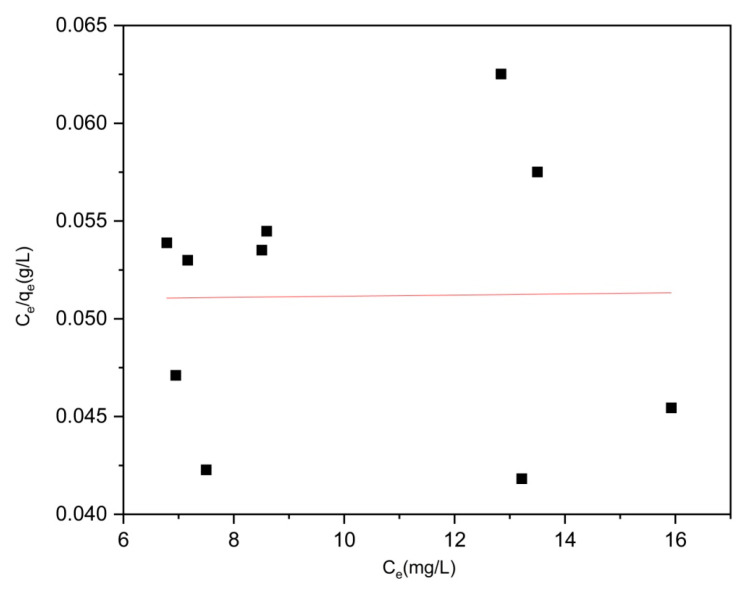
Langmuir isotherm of moxifloxacin onto Co/Cd-MOF carbon material.

To validate the experimental findings regarding moxifloxacin adsorption by Co/Cd-MOF carbon materials, we employed the Langmuir and Freundlich isotherm models to analyze the collected data (refer to Figure 11 and Figure 12 and Table 3). The obtained R^2^ values for the Langmuir and Freundlich models were 0.1247 and 0.8361, respectively. Importantly, the Freundlich model exhibited superior agreement with the actual moxifloxacin adsorption process, suggesting a multilayer adsorption mechanism for moxifloxacin on Co/Cd-MOF carbon materials.

**Figure 13:**
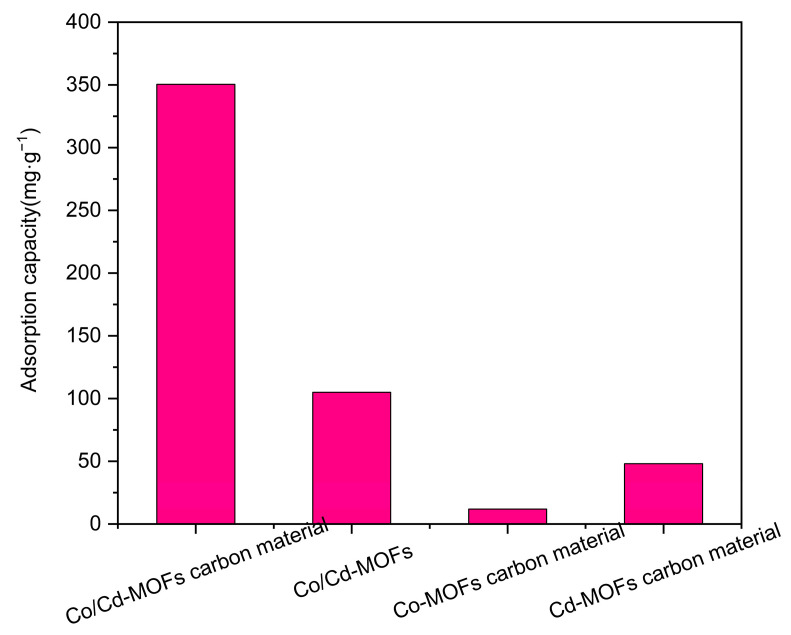
Comparison of adsorption capacity of moxifloxacin.

In this study, a comparison was conducted between the carbon materials derived from Co/Cd-MOFs and the original Co/Cd-MOFs themselves. The adsorption capacity of moxifloxacin in a 50 ppm solution was evaluated after introducing Co/Cd-MOFs, followed by their respective carbonization processes including Co-MOF carbonization and Cd-MOF carbonization. Figure 13 illustrates the adsorption performance within a 5 h timeframe. It can be observed from the figure that the process of carbonization enhances the exposure of active sites, resulting in improved moxifloxacin adsorption in the resultant carbonized Co/Cd-MOF material.

A comparison of experimental results often necessitates evaluating them in relation to other adsorbents. Table 4 presents the adsorption capacities of various adsorbents for moxifloxacin, revealing that the Co/Cd-MOF carbon material demonstrates superior efficacy in adsorbing moxifloxacin. Chai et al. employed chromic nitrate hydrate and terephthalic acid to synthesize MIL-101, achieving a moxifloxacin adsorption capacity of 86 mg/g at 220 °C. In contrast, Zhao et al. utilized ZrOCl_2_·8H_2_O and H_3_BTC to prepare MOF-808-AA and MOF-808-SIPA, resulting in significantly higher moxifloxacin adsorption capacities of 174.6 mg/g and 287.1 mg/g [40,41], respectively. The variation in adsorption amounts can be attributed to the distinct metal–ligand structures formed by different metal ions.

**Figure 14:**
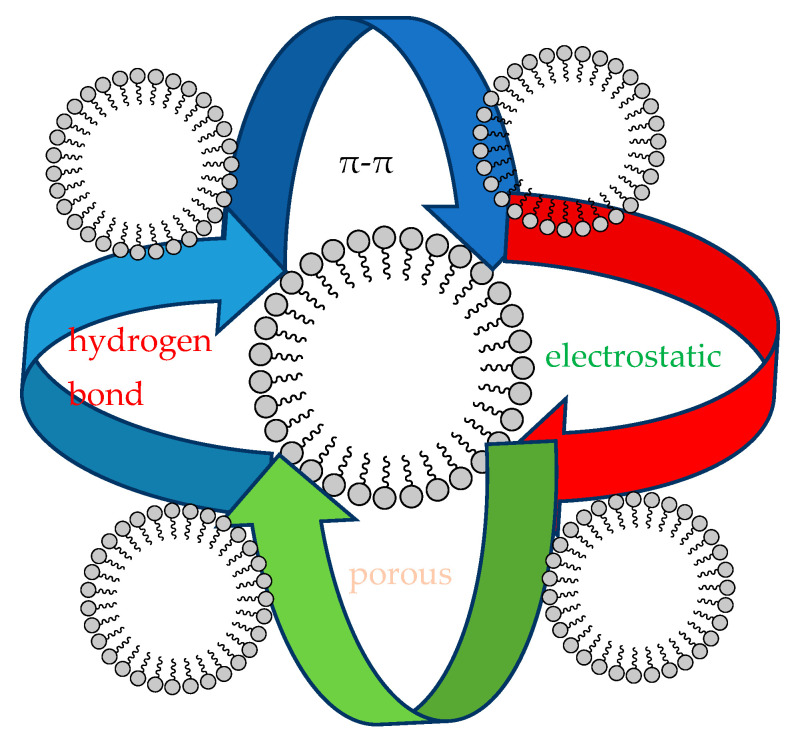
Adsorption mechanism of moxifloxacin by Co/Cd-MOFs.

To sum up, this can mainly be attributed to the mechanism shown in Figure 14. Firstly, both moxifloxacin and Co/Cd-MOF carbon materials possess a benzene ring, enabling them to adhere together through π-π bonding. Secondly, the significant specific surface area and pore size of the Co/Cd-MOF carbon material facilitate its ability to effectively adsorb moxifloxacin through these pores. Thirdly, the presence of group electronegativity on the surface of the Co/Cd-MOF carbon material allows for net charge-based adhesion with moxifloxacin. Lastly, hydrogen bonds may also contribute to the binding between Co/Cd-MOF carbon materials and moxifloxacin. Collectively, these aforementioned factors contribute significantly to the excellent performance exhibited by Co/Cd-MOF carbon materials in terms of adsorbing moxifloxacin.

To evaluate the influence of temperature on moxifloxacin adsorption by Co/Cd-MOF carbon materials, a solution containing 50 ppm of moxifloxacin was treated with 20 mg of Co/Cd-MOF carbon materials. The impact of different temperatures (T = 10 °C, 30 °C, 50 °C) on the interaction between Co/Cd-MOF carbon materials and moxifloxacin during the adsorption process was investigated in this study. The experimental procedure can be summarized as follows:

(3) $${\mathrm{lnK}}_{0}=\frac{\Delta S^{0}}{R}-\frac{\Delta H^{0}}{\mathrm{RT}}$$

(4) $$\Delta G^{0}=-{\mathrm{RTlnK}}_{0}$$

(5) $$K_{0}=\frac{q_{e}}{c_{e}}$$

For the purpose of this study, we employed the ideal gas constant (R = 8.314 J mol^−1^ K^−1^) and Langmuir adsorption constant (K_0_ in L/mol). To determine the values of ΔH^0^ and ΔS^0^, a van’t Hoff plot was utilized by performing linear regression analysis on lnK0 against 1/T. Specifically, ΔH^0^ was calculated as the negative slope multiplied by R, while ΔS^0^ was obtained by multiplying the intercept with R.

**Figure 15:**
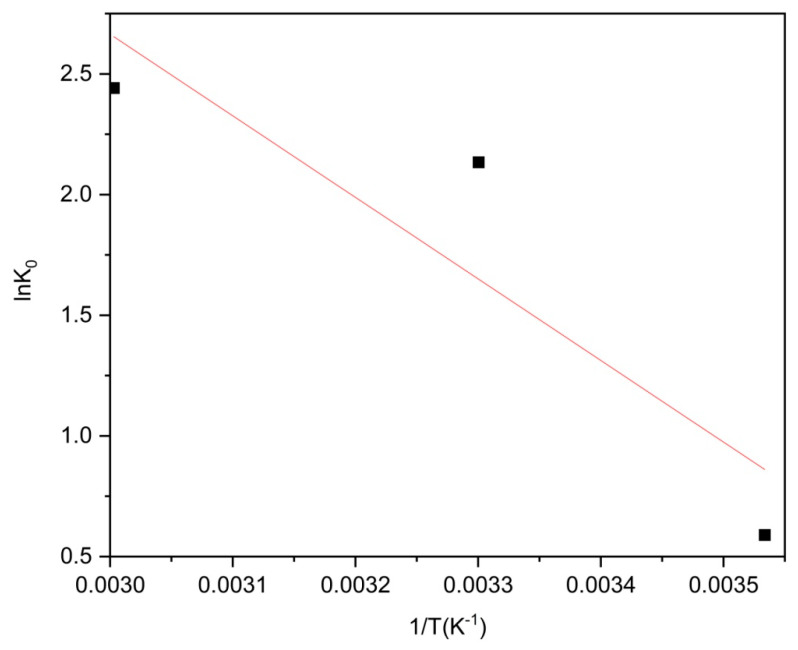
Van’t Hoff plots to obtain the ΔH and ΔS of moxifloxacin adsorption over Co/Cd-MOF carbon materials.

The experimental results presented in Figure 15 and Table 5 provide compelling evidence supporting the exothermic and spontaneous adsorption of moxifloxacin by Co/Cd-MOF carbon materials. This is further supported by the observation of negative values for ΔG^0^ and positive values for both ΔH^0^, indicating a favorable energetic process. The adsorption mechanism involves a combination of physical and chemical processes, where enthalpy changes ranging from 84 to 420 kJ/mol primarily arise from chemisorption. Instances where enthalpy changes are below 84 kJ/mol suggest physical adsorption [42]. Therefore, it can be inferred that the predominant mode of moxifloxacin adsorption on Co/Cd-MOF carbon materials is predominantly physical in nature. Consequently, alterations in entropy and enthalpy significantly impact the adsorptive behavior of moxifloxacin on Co/Cd-MOF carbon materials [43]. Physical interactions serve as the primary driving force behind the process of adsorption.

## 3. Experiment

### 3.1. Experimental Material

In the experiment, moxifloxacin provided by Shanghai McLean Biochemical Technology Co., Ltd. (Shanghai, China) was selected as the target antibiotic, while the organic ligand 1,3,5-benzoic acid was procured from Shanghai McLean Biochemical Technology Co., Ltd. Additionally, cadmium acetate dihydrate and cobalt chloride hexahydrate were employed as metal ion sources obtained from Shanghai Haohong Biomedical Technology Co., Ltd. (Shanghai, China) and Shanghai Aladdin Biochemical Technology Co., Ltd. (Shanghai, China). The deionized water utilized in the experiment was prepared on-site as required.

### 3.2. Preparation of Co/Cd-MOF Carbon Materials

Three separate 50 mL small beakers were used to individually contain weighed amounts of 1,3,5-benzoic acid (0.0015 mol, 0.3152 g), cadmium acetate dihydrate (0.0015 mol, 0.3998 g), and cobalt chloride hexahydrate (0.0015 mol, 0.3569 g). These substances were then dissolved in 30 mL of dimethylformamide (DMF) as the organic solvent using an ultrasonic cleaning machine. The resulting solutions were transferred to a reaction kettle and subjected to constant temperature drying at a temperature of 120 °C for a duration of 14 h. Once the reaction was complete, the mixture was cooled down to room temperature and filtered before being washed three times with DMF and distilled water in order to obtain the products known as Co/Cd-MOFs. The prepared Co/Cd-MOFs were collected using a small crucible and underwent high-temperature carbonization in a muffle furnace by gradually increasing the temperature at a rate of 5 °C/min until reaching a final temperature of 900 °C followed by cooling down over 2 h back to room temperature.

### 3.3. The Reconstitution of an Aqueous Solution of Moxifloxacin

**Figure 16:**
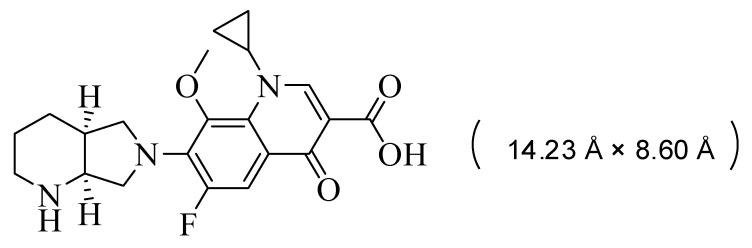
A structural diagram depicting the molecular composition of moxifloxacin.

In order to investigate the properties of Co/Cd-MOF carbon materials, moxifloxacin was selected as the target molecule in this study (the structure and size of moxifloxacin [40] are illustrated in Figure 16). A total of 100 mg of moxifloxacin was weighed and transferred into a 1000 mL beaker, followed by stirring on a magnetic stirrer for one hour after five hours of ultrasound treatment. Subsequently, the mixture was transferred to a volumetric bottle with a fixed volume of 1000 mL, and it was prepared as required.

### 3.4. Removal of Moxifloxacin from Wastewater by Co/Cd-MOF Carbon Materials

In order to investigate the adsorption performance of Co/Cd-MOF-based carbon materials on moxifloxacin antibiotics, various quantities (20 mg, 30 mg, 40 mg, 50 mg, 100 mg) of Co/Cd-MOF carbon materials were added to a solution containing different concentrations (20 mg/L, 30 mg/L, 40 mg/L, 50 mg/L) of moxifloxacin (200 mL) and stirred under natural light. At intervals of every 30 min, samples were collected, and the concentration of moxifloxacin in the solution was analyzed using an ultraviolet spectrometer. The calculation formula used is as follows:

q_e_ = ((C_0_ − C_e)_V)/m (6)

Removal rate (%) = ((C_0_ − C_t)_)/C_0_ × 100% (7)

In this study, we took into account moxifloxacin’s initial concentration (C_0_) (mg/L), its concentration at adsorption equilibrium (C_e_) (mg/L), the concentration at time t (C_t_) (mg/L), the solution volume (V) (L), and the mass of Co/Cd-MOF-based carbon materials (m) (mg).

## 4. Conclusions

In this study, carbon materials derived from Co/Cd-MOFs were successfully synthesized and characterized using techniques such as FT-IR, XRD, SEM, etc. Subsequently, these characterized materials were utilized for the efficient removal of moxifloxacin. The experimental results demonstrate that a remarkable maximum adsorption capacity of 350.4 mg/g was achieved when employing a mass of 20 mg for the Co/Cd-MOF-derived carbon material in a moxifloxacin solution concentration of 50 ppm. Furthermore, with an increased mass of 100 mg for the Co/Cd-MOF-derived carbon material while maintaining the moxifloxacin solution concentration at 50 ppm, an impressive removal rate of 88.9% was attained within just 5 h. Kinetic modeling and isothermal adsorption studies reveal that the adsorption process followed the pseudo-second-order kinetic model and Freundlich isotherm equation, respectively. The analytical findings suggest that physical adsorption predominantly contributes to removing moxifloxacin using Co/Cd-MOF-derived carbon materials as effective sorbents. A comparative analysis with similar adsorbents highlights the superior performance of Co/Cd-MOF-derived carbon materials in terms of their moxifloxacin removal capabilities. Therefore, these findings underscore the significant theoretical potential offered by Co/Cd-MOF-derived carbon materials for environmental remediation applications targeting moxifloxacin.

## Figures and Tables

**Table 1:** Kinetic parameters for the adsorption of moxifloxacin over the Co/Cd-MOF carbon materials (linear).

Concentration	Mass	PSO Model		PFO Model		
		K_2_ (g·(mg·min)^−1^)	R^2^	K_1_ (L·min^−1^)	R^2^	q_max,exp_
20	20	0.0105	0.9922	0.0004	0.3891	95.00
	30	0.0179	0.9940	0.0012	0.7283	47.40
	40	0.0206	0.9933	0.0011	0.9787	44.70
	50	0.0118	0.9904	0.0044	0.9966	72.70
30	20	0.0124	0.9909	0.0004	0.3478	86.60
	30	0.0133	0.9978	0.0009	0.8575	59.20
	40	0.0177	0.9822	0.0009	0.9713	47.70
	50	0.0164	0.9862	0.0012	0.9900	49.90
40	20	0.0051	0.9933	0.0014	0.9569	115.0
	30	0.0065	0.9900	0.0019	0.9419	92.00
	40	0.0049	0.98769	0.0048	0.9626	130.50
	50	0.0048	0.9939	0.0062	0.9934	109.20
50	20	0.0029	0.9904	0.0028	0.9698	253.00
	30	0.0054	0.9868	0.0030	0.4664	167.20
	40	0.0042	0.99365	0.0038	0.9542	134.90
	50	0.0039	0.99481	0.0052	0.9955	126.60

**Table 2:** Kinetic parameters for the adsorption of moxifloxacin over the Co/Cd-MOF carbon materials (nonlinear).

Concentration	Mass	PSO Model		PFO Model	
		K _2_(g·(mg·min)^−1^)	R^2^	K _1_(L·min^−1^)	R^2^
20	20	-	0.9953	-	0.1855
	30	-	0.9987	-	0.9930
	40	-	0.9983	-	0.9725
	50	-	0.9993	-	0.9956
30	20	-	0.9907	-	0.8193
	30	-	0.9984	-	0.9951
	40	-	0.9964	-	0.9676
	50	-	0.9954	-	0.9903
40	20	-	0.9933	-	0.9918
	30	-	0.9921	-	0.9950
	40	-	0.9844	-	0.9851
	50	-	0.9920	-	0.9951
50	20	-	0.9916	-	0.9861
	30	-	0.9839	-	0.7844
	40	-	0.9952	-	0.9896
	50	-	0.9931	-	0.9980

**Table 3:** Summary of isotherm constants for removal of moxifloxacin by Co/Cd-MOF carbon material.

Langmuir Isotherm		Freundlich Isotherm		Sips		Brouers–Sotolongo	Uncertainty	
K	R^2^	n	R^2^	R^2^	k	R^2^		
2.9061	0.1247	1.0070	0.8361	0.8156	20.8016	0.7829	±2%	

**Table 4:** Comparison of maximum adsorption capacities of various adsorbents for moxifloxacin.

Adsorbent	q_max_ (mg g^−1^)	Reference
Co/Cd-MOF carbon material	350.4	This work
Co/Cd-MOFs	105.0	
MIL-101	86.0	[40]
MOF-808-SIPA	287.1	[41]
MOF-808-AA	174.6	

**Table 5:** The thermodynamic parameters of moxifloxacin over the Co/Cd-MOF carbon material.

T (K)	ΔG^θ^ (kJ/mol)	ΔH^θ^ (−slope × R)(KJ/mol)	S^θ^ (intercept × R) (J/mol/K)
293	−3.1	28.1	106.5

## Data Availability

The data that support the findings of this study are available from the corresponding author upon reasonable request.
